# Factors Affecting Prognosis in Patients with Extramedullary Myeloma: A Single-Center Experience

**DOI:** 10.3390/jcm15176765

**Published:** 2026-08-31

**Authors:** Ersin Bozan, Samet Yaman, Gökcen Bozan, Mahmut Esad Durmuş, Burcu Aslan Candır, Uğur Hatipoğlu, Barış Boral, Dicle İskender, Bahar Uncu Ulu, Tuğçe Nur Yiğenoğlu, Mehmet Sinan Dal, Fevzi Altuntaş

**Affiliations:** 1 Hematology and Bone Marrow Transplantation Unit, Ankara Oncology Training and Research Hospital, Health Sciences University, Ankara 06170, Turkey; drsametyaman@hotmail.com (S.Y.); drburcuaslancandir@gmail.com (B.A.C.); ugurugur2192@gmail.com (U.H.); diclekoca@yahoo.com (D.İ.); baharuncu@gmail.com (B.U.U.); dr.nuryigenoglu@gmail.com (T.N.Y.); dr.sinandal@gmail.com (M.S.D.); faltuntas@hotmail.com (F.A.); 2 Internal Medicine Clinic, Ankara Oncology Training and Research Hospital, Health Sciences University, Ankara 06170, Turkey; gokcennarslan@gmail.com (G.B.); esad.durmus04@gmail.com (M.E.D.); 3 Immunology Department, Ankara Oncology Training and Research Hospital, Health Sciences University, Ankara 06170, Turkey; boralbaris@gmail.com

**Keywords:** extramedullar myeloma, prognostic factors, biomarkers

## Abstract

**Background/Objectives**: In multiple myeloma, extramedullary involvement confers poor prognosis. Extramedullary involvement in multiple myeloma may manifest at the time of diagnosis or during a relapse, and it represents a severe condition. Patients with bone involvement are referred to as EM-B, while those with soft tissue involvement are referred to as EM-S. Although various studies have been conducted, the number of studies that categorize extramedullary involvement and examine the effects of flow cytometry, treatment, and genetic findings on prognosis is quite limited. We aimed to contribute to the literature by addressing this gap. **Methods**: In our study, we retrospectively examined the data of 281 patients followed at our center between 2011 and 2023. Clinical/laboratory data and flow cytometry profiles were collected. Survival was analyzed using Kaplan–Meier and Cox models; logistic regression was used for categorical variables. Hazard ratios (HRs), odds ratios (ORs), and 95% confidence intervals (CIs) were calculated; *p*-values were reported to three decimals. **Results**: Extramedullary involvement was absent in 134 (47.7%) patients, while involvement was detected in 147 (52.3%) patients. The median age of the patients was 60 years, and the median follow-up period was 35.4 months. No association was found between CD56 expression on plasma cells, higher Revised Multiple Myeloma International Staging System (R-ISS) score, and extramedullary involvement. Overall survival (OS) was found to be significantly lower in patients with extramedullary involvement. **Conclusions**: The findings of our study are consistent with many studies in the literature, supporting the finding that survival rates are lower in myeloma patients with extramedullary involvement. Further studies are needed to elucidate the pathogenesis in patients with extramedullary involvement in multiple myeloma and to provide a basis for targeted therapies. Studies involving immunotherapy and cellular therapies may offer a new avenue for these patients.

## 1. Introduction

Multiple myeloma is a malignancy caused by the uncontrolled proliferation of monoclonal plasma cells. After Non-Hodgkin lymphomas, it is the second most common hematological malignancy. Myeloproliferative neoplasms and chronic lymphocytic leukemia/small lymphocytic lymphoma (CLL/SLL) are other frequently observed malignancies [1]. Extramedullary involvement of myeloma has been reported in 15–20% of newly diagnosed patients [2]. Extramedullary disease (EMD) in multiple myeloma is primarily categorized according to its association with osseous structures, temporal origin, and tissue engagement [3]. In our study, we classified extramedullary multiple myeloma based on its relationship with bone.

Extramedullary myeloma-bone (EM-B) refers to bone-only disease, while extramedullary myeloma-soft tissue (EM-S) indicates soft tissue myeloma, which would be located in the gastrointestinal tract, lungs, or upper aerodigestive system [3]. Extramedullary dissemination of the disease is now diagnosed in the earlier course of myeloma, mainly due to advanced imaging studies [4]. From a clinicopathological perspective, EM-B and EM-S can exhibit differences across a range of parameters, such as site of involvement, tumor burden, laboratory findings, hematologic-cytogenetic findings, and response to treatment. Mostly, individuals with EM-S exhibit a more severe disease manifestation, increased tumor load, and poorer survival rates relative to those with EM-B in multiple myeloma [5].

Strong evidence for EM prognosis, treatment, and correlation with other disease features and prognostic indices is quite limited. In general, studies have shown that patients with extramedullary multiple myeloma experience sub-optimal response rates and have a dismal prognosis [5]. Due to the limited number of studies conducted, there is no established standard treatment for patients with extramedullary myeloma, unlike solitary plasmacytoma. The relationship between CD56 expression and extramedullary involvement is not clear, while studies have yielded inconsistent results [6,7]. Furthermore, the revised version of the International Staging System (R-ISS) has limited sensitivity in extramedullary myeloma [8].

In this study, we aimed to examine the prognosis, surface markers, and their relationship with laboratory values, genetic risk factors, and clinical course of a total of 281 patients diagnosed with multiple myeloma, 147 of whom had extramedullary myeloma.

## 2. Materials and Methods

This single-center, retrospective, and non-randomized observational study was conducted with the approval of the Dr. Abdurrahman Yurtaslan Oncology Training and Research Hospital Ethics Committee (Approval No: 2023-04/41) and adhered to the principles outlined in the Declaration of Helsinki.

### 2.1. Study Design and Patients

A total of 281 newly diagnosed multiple myeloma patients followed at our center between 2011 and 2023 were screened. International Myeloma Working Group (IMWG) multiple myeloma diagnostic criteria were used for the myeloma diagnosis [9]. All patients aged 18 and older were included. Patients with incomplete clinical records or concurrent hematologic malignancies were excluded.

Demographic data, blood type, complete blood counts and biochemistry panel at diagnosis, Eastern Clinical Oncology Group (ECOG) performance score, heavy and light chain subtypes, ISS and R-ISS stages, EM disease status, and comorbidities were recorded.

Patients were treated with first-line bortezomib/cyclophosphamide/dexamethasone (VCD), bortezomib/dexamethasone (VD), or bortezomib/adriamycin/dexamethasone (VAD) due to national reimbursement requirements and the standard operating procedure of our clinic. Autologous hematopoietic stem cell transplant (ASCT) was also standard for eligible patients.

Routine immunophenotyping of bone marrow samples was performed using flow cytometry (Becton Dickinson FACS Lyric, Franklin Lakes, NJ, USA) in the Immunology Laboratory of Ankara Oncology Hospital. Samples were prepared and incubated with fluorescently labeled monoclonal antibodies per the manufacturer’s guidelines. Flow cytometry analyses were conducted using BD FACSuite software (version 1.5) and verified by pathologists and specialists. CD56 positivity was defined as expression in at least 20% of plasma cells. Clonal plasma cells were identified based on their characteristic CD38/CD138 expression pattern and light chain restriction.

### 2.2. Definitions

EM-B and EM-S are distinct patterns of EM involvement: EM-B is classically defined as myeloma with bone plasmacytomas, whereas EM-S is composed of soft-tissue-dwelling plasmacytomas and medullary disease [3]. New diagnoses were made based on IMWG criteria. IMWG criteria were used for response assessment (Table 1 and Table 2) [10]. The Eastern Cooperative Oncology Group (ECOG) performance status is a standardized scale used to assess a patient’s functional status and ability to perform daily activities, ranging from 0 (fully active) to 4 (completely disabled) [11]. Progression-free survival (PFS) was estimated as the time between the first treatment and relapse, death, or last contact. Overall survival (OS) was defined as the time between the first treatment and death or last contact.

### 2.3. Endpoints

The primary endpoint of the study was OS. Secondary endpoints were selected as; PFS, CD56 positivity in EM.

### 2.4. Statistical Analysis

Statistical analyses were performed using IBM SPSS Statistics version 29 (Armonk, NY, USA). Statistical significance was set at *p* < 0.05. Patient characteristics were summarized as medians (min–max), means and standard deviations for continuous variables, and frequencies (%) for categorical variables. Univariate and multivariate logistic regression models were applied to identify factors influencing treatment response. Results are presented as odds ratios (OR) with 95% confidence intervals (CI). Kaplan–Meier survival curves were generated to evaluate progression-free survival (PFS) and overall survival (OS). Log-rank tests were used to compare survival distributions. Cox proportional hazards regression models were utilized to identify independent predictors of survival. Results are reported as hazard ratios (HR) with 95% CI.

## 3. Results

A total of 281 multiple myeloma patients were included in the study. The median age of the patients was 60 years, and the median follow-up period was 35.4 months. Demographic and clinical characteristics of the patients are summarized in Table 3 and Table 4.

The median overall survival (OS) could not be reached during the study period (Table 5).

**Figure 1:**
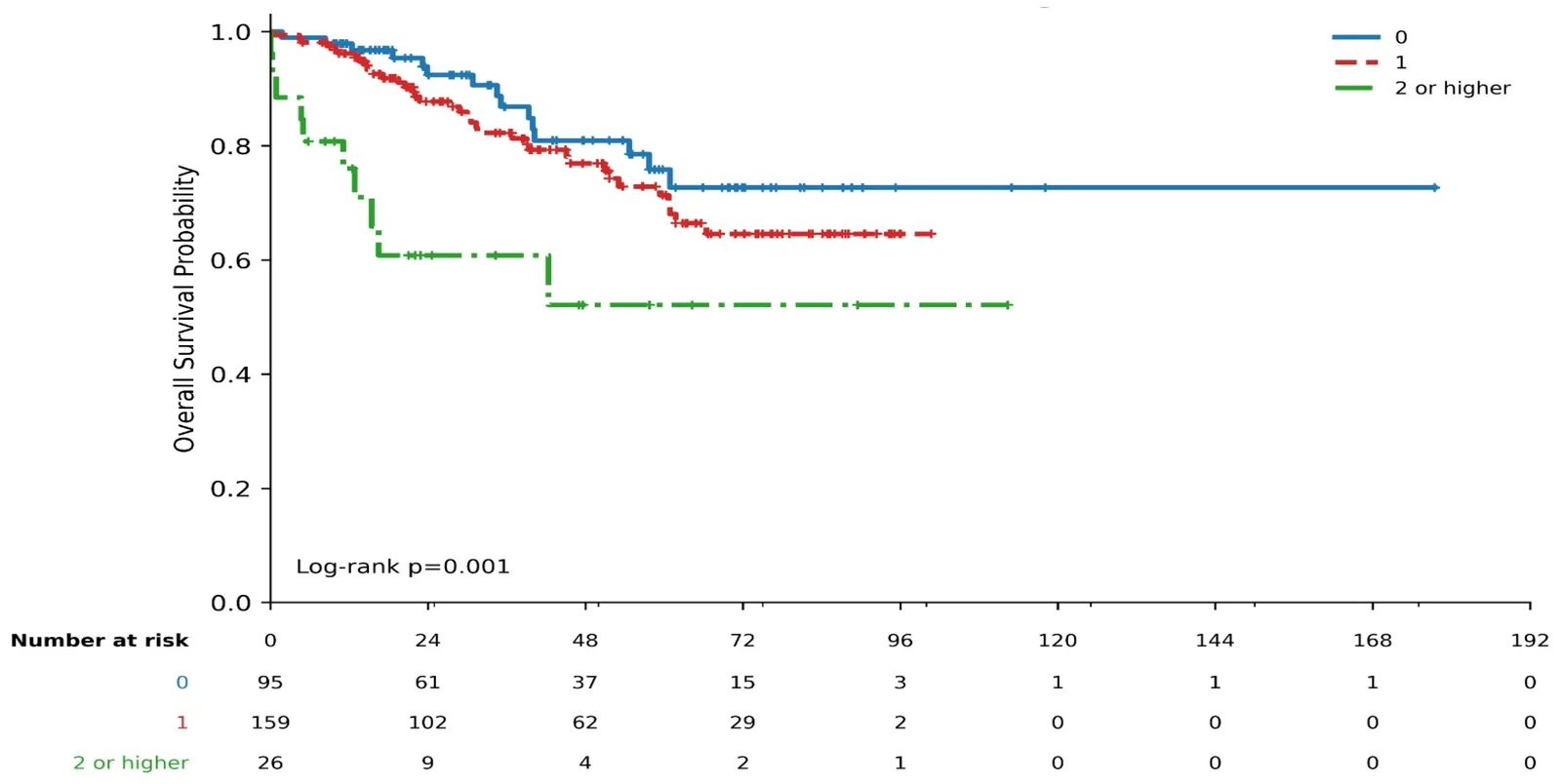
Overall survival according to ECOG.

**Figure 2:**
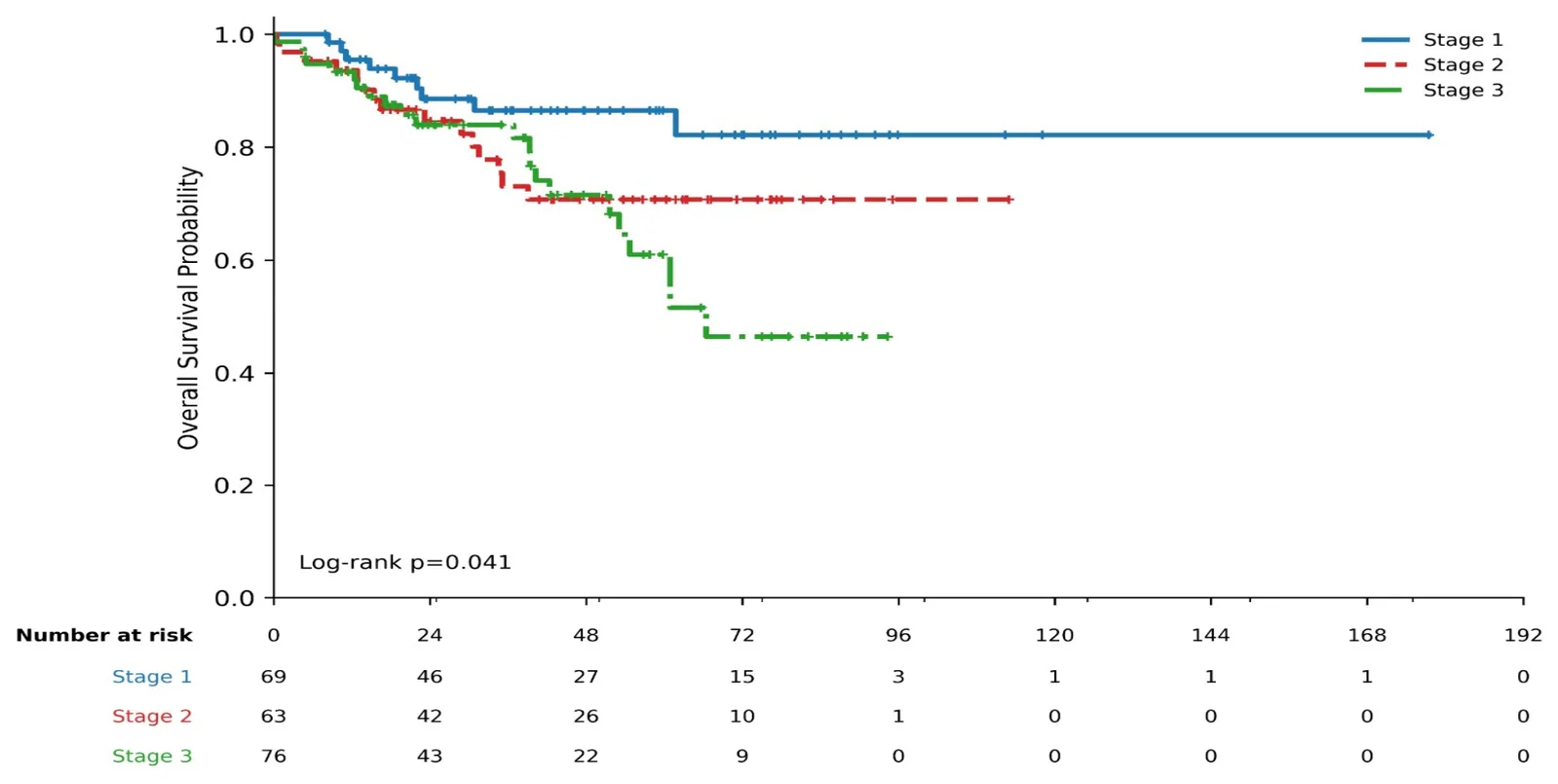
Overall survival according to ISS stage.

Advanced ECOG performance score was associated with worse prognosis (Figure 1) (*p* < 0.001). Similarly, patients with higher ISS score had significantly worse prognosis (Figure 2) (*p* = 0.041).

**Figure 3:**
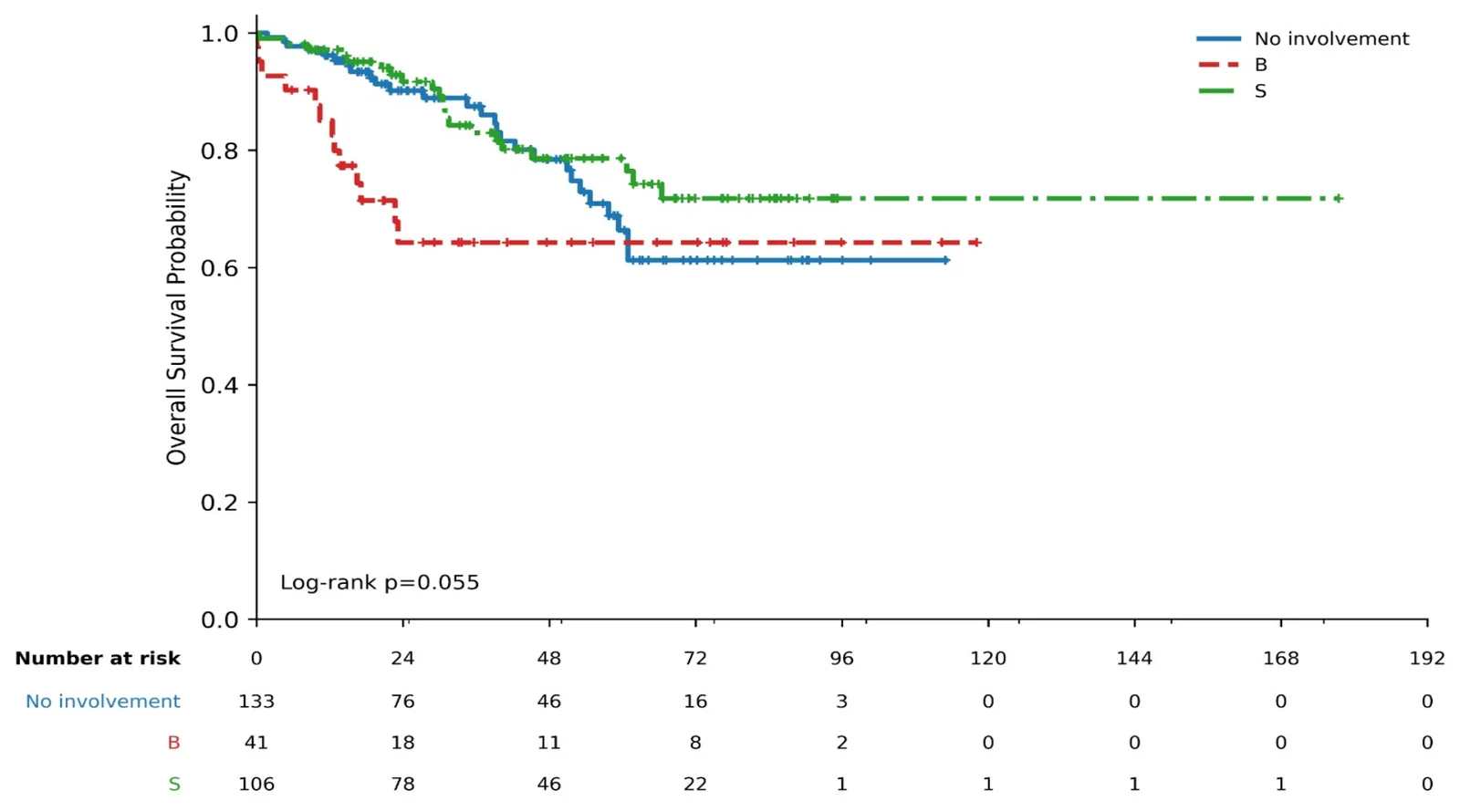
Overall survival according to involvement.

OS was significantly lower in patients with extramedullary involvement (Figure 3) (*p*: 0.049).

**Figure 4:**
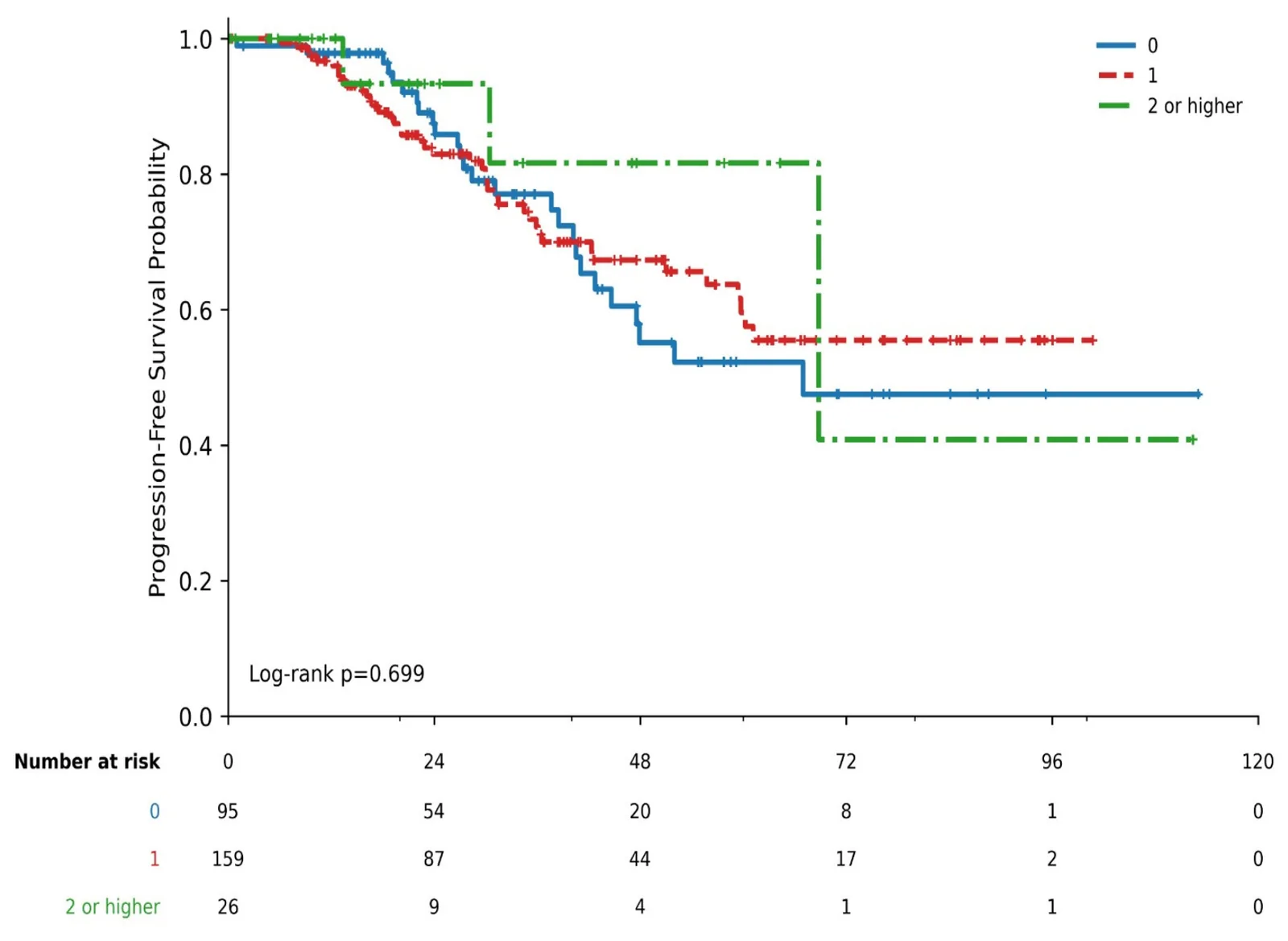
Progression-Free survival according to ECOG.

**Figure 5:**
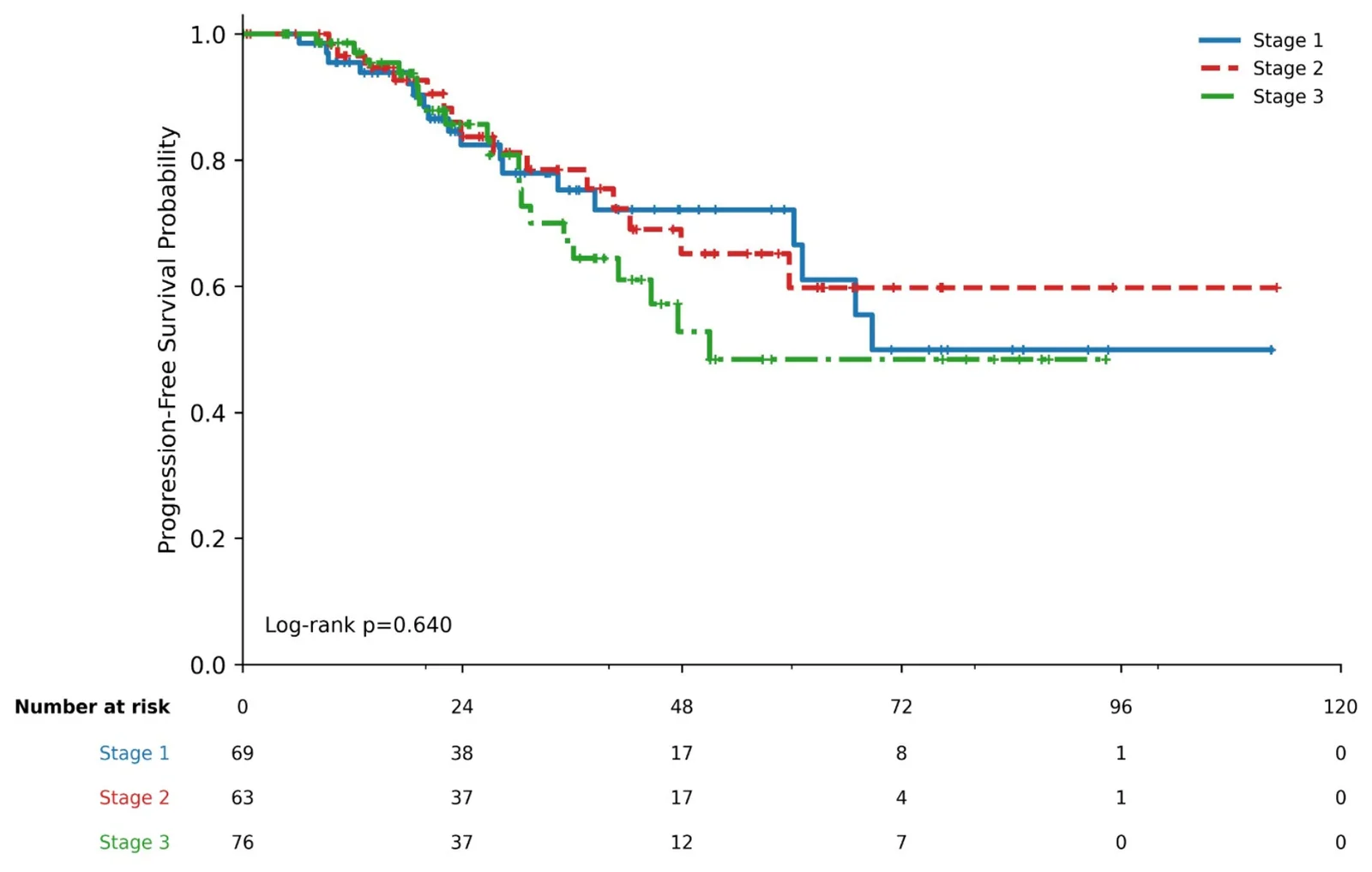
Progression-Free survival according to ISS stage.

**Figure 6:**
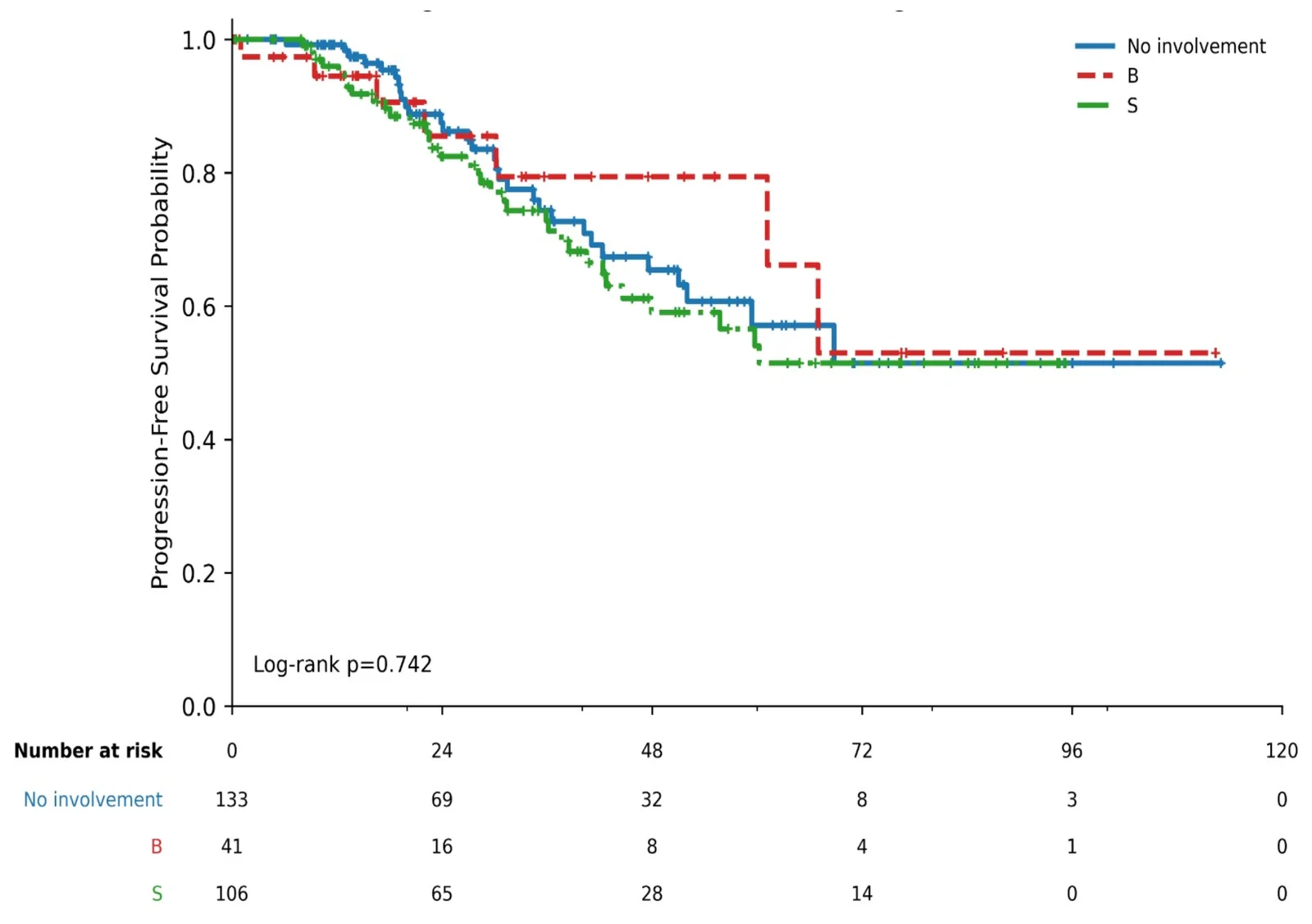
Progression-Free survival according to involvement.

When patient PFS was evaluated, the median PFS (months) could not be reached (Table 6). The median PFS (months) was not found to be statistically significant according to the variables (Figure 4, Figure 5 and Figure 6) (*p* > 0.05).

As shown in Table 5, ECOG, ISS stage, and extramedullary involvement variables were found to be significant in univariate analyses. Variables found to be significant in univariate analyses were included in the multivariate Cox regression model (Table 7). According to the model results, having an ECOG of 2 or higher increased the risk of death 3.78 times (HR: 3.78, 95% CI: 1.54–9.28, *p* = 0.004), having ISS Stage-3 increased the risk 2.93 times (HR: 2.93, 95% CI: 1.30–6.62, *p* = 0.010), and having extramedullary involvement increased the risk 2.46 times (HR: 2.46, 95% CI: 1.17–5.17, *p* = 0.017).

The relationship between CD56 expression and extramedullary involvement was investigated, and no statistically significant results were obtained (Table 8). (*p* = 0.281).

## 4. Discussion

Our study showed (i) lower OS but similar PFS between EM and non-EM, (ii) no significant association of flow cytometric CD56 expression between EM and non-EM.

Studies in the literature have shown that patients with extramedullary involvement at the time of diagnosis or after recurrence have a worse prognosis [5,12]. A retrospective study by Çiftçiler et al. demonstrated that the 5-year OS rate was significantly lower in patients presenting with either EM-B or EM-S compared to those without any plasmacytoma presentation [5]. This inferior prognostic impact is further justified by a large-scale, multi-institutional study conducted by Beksac et al., which evaluated 226 myeloma patients across the Balkan Myeloma Study Group and Barcelona University. They reported that, even within the era of novel therapies, true extramedullary organ/tissue plasmacytomas carry a highly aggressive clinical course. In their cohort, patients with EM-S at initial diagnosis achieved a median OS of only 46.5 months, whereas the median OS for the paraosseous EM-B group was not reached [12]. In our study, consistent with the literature, patients with extramedullary involvement had a lower overall survival rate and a significantly higher risk of death. No significant difference was found in PFS among the three groups: EM-B, EM-S, and those without plasmacytoma.

Unsurprisingly, in our study, ISS stage and elevated ECOG were associated with a worse prognosis.

There are studies that hypothesize that CD56 expression, which ensures the fixation of plasma cells to the stromal structure, may lead to more frequent extramedullary involvement and have prognostic significance when deficient. In our study, similar to the studies by Ceran et al., no association was found between CD56 expression and extramedullary involvement [13]. Studies in the literature suggest that CD56 negativity is more frequently associated with extramedullary involvement [14]. In our study, we did not find an association between CD56 expression and prognosis.

The importance of surface markers in targeted therapies is increasing day by day. Identifying such a specific target in patients with extramedullary myeloma could offer great hope for treatment. Further research in this direction could be significant in terms of making progress in treatment. At least in our study, we concluded that CD56 is not currently such a target.

The lack of a close association between R-ISS and extramedullary myeloma may indicate that factors other than chromosomal abnormalities play a role in extramedullary involvement.

One of the limitations of our study is that not all patients received the same treatment. The efficacy of novel agents such as lenalidomide, selinexor, pomalidomide, and elranatamab could not be evaluated in our study, which constitutes another limitation. The number of studies investigating the efficacy of novel treatment modalities on extramedullary myeloma is quite limited and contradictory [15,16]. In the new era of myeloma, more studies with longer follow-up periods and larger patient numbers are needed to investigate the efficacy of many novel agents that can be added to treatment for extramedullary myeloma.

## 5. Conclusions

This study shows that the prognosis is worse in patients with extramedullary myeloma even after autologous stem cell transplantation. Higher tumor burden, genetic mutation differences, and inherently aggressive clones independent of the bone marrow may be associated with a poor prognosis. Further studies involving more patients and multiple centers to determine the association between different surface markers and genetic mutations and extramedullary myeloma appear to be crucial for elucidating the pathogenesis and offering hope for treatment.

## Figures and Tables

**Table 1:** IMWG criteria for the diagnosis of MM.

Clonal bone marrow plasma cells ≥ 10% or a biopsy-proven bony or extramedullary (soft tissue) plasmacytoma.
Myeloma-defining events (Any 1 Required) 1. CRAB Features (End-Organ Damage) C—Hypercalcemia: Serum calcium > 1 mg/dL (>0.25 mmol/L) higher than the upper limit of normal, or absolute value > 11 mg/dL (>2.75 mmol/L). R—Renal insufficiency: Serum creatinine > 2 mg/dL (>177 µmol/L) or creatinine clearance < 40 mL/min. Anemia: Hemoglobin value < 10 g/dL (100 g/L) or >2 g/dL (20 g/L) below the normal lower limit. B—Bone lesions: One or more osteolytic (bone-destroying) lesions on skeletal radiography, CT, or PET-CT. 2. Biomarkers of Malignancy (SLiM) S—Sixty percent clonal plasma cells: Clonal bone marrow plasma cell percentage ≥ 60%. Li—Light chain ratio: Involved-to-uninvolved serum free light chain (sFLC) ratio ≥ 100 (provided the involved light chain is at least 100 mg/L). M—MRI focal lesions: More than one focal lesion (at least 5 mm in size) on MRI studies.

IMWG, International Myeloma Working Group; MRI, magnetic resonance imaging.

**Table 2:** IMWG response criteria.

Response	IMWG Criteria
sCR	CR as defined below plus normal FLC ratio and absence of clonal cells in bone marrow by immunohistochemistry or immunofluorescence
CR	Negative immunofixation on the serum and urine and disappearance of any soft tissue plasmacytomas and <5% plasma cells in BM
VGPR	Serum and urine M protein detectable by immunofixation but not on electrophoresis or >90% reduction in serum M protein plus urine M protein level < 100 mg/24 h
PR	>50% reduction in serum M protein and reduction in 24 h urinary M protein by >90% or to <200 mg/24 h; if the serum and urine M protein are unmeasurable, a >50% decrease in the difference between involved and uninvolved FLC levels is required in place of the M protein criteria
	If serum and urine M protein are not measurable, and serum free light assay is also not measurable, >50% reduction in plasma cells is required in place of M protein, provided baseline BM plasma cell percentage was >30%
	In addition to the above listed criteria, if present at baseline, a >50% reduction in the size of soft tissue plasmacytomas is also required
SD	Does not meet criteria for sCR, CR, VGPR, PR, or PD
PD	≥25% increase in M-protein, urine protein, or plasma cells; new lesions or clear progression of existing lesions

CR, complete response; FLC, free light chain; h, hours; IMWG, International Myeloma Working Group; M, monoclonal; PR, partial response; sCR, stringent complete response; VGPR, very good partial response; SD, stable disease; PD, progressive disease.

**Table 3:** Distributions of sociodemographic and clinical variables.

Variables	N/%
Age	
Mean ± SD	60.25 ± 10.69
Median (min–max)	60.0 (28–83)
≤65	226/281 (80.4%)
>65	55/281 (19.6%)
Gender	
Men	172/281 (61.2%)
Women	109/281 (38.8%)
Blood type	
A	134/281 (47.7%)
B	45/281 (16.0%)
AB	26/281 (9.3%)
0	76/281 (27.0%)
ECOG	
0	95/281 (33.8%)
1	160/281 (56.9%)
2	22/281 (7.8%)
3	3/281 (1.1%)
4	1/281 (0.4%)
Immunoglobulin subtype	
Ig A kappa	25/248 (10.1%)
Ig A lambda	16/248 (6.5%)
Ig G kappa	88/248 (35.5%)
Ig G lambda	56/248 (22.6%)
Kappa Light Chain	46/248 (18.5%)
Lambda Light Chain	17/248 (6.9%)
ISS Stage	
Stage-1	69/209 (33.1%)
Stage-2	63/209 (30.1%)
Stage-3	77/209 (36.8%)
R-ISS Stage	
Stage-1	54/155 (34.8%)
Stage-2	72/155 (46.5%)
Stage-3	29/155 (18.7%)
EM involvement	
MM with no plasmacytoma	134/281(47.7%)
EM-B	41/281 (14.6%)
EM-S	106/281 (37.7%)
Comorbidity	
Negative	143/281 (50.9%)
Positive	138/281 (49.1%)
Baseline CD56 positivity	
Negative	38/281 (13.5%)
Positive	165/281 (58.7%)
Unknown	78/281 (27.8%)
Progression	
Negative	209/281 (74.4%)
Positive	72/281 (25.6%)
Mortality	
Alive	219/281 (77.9%)
Exitus	62/281 (22.1%)
Follow-up Period (Months)	
Mean ± SD	41.05 ± 28.32
Median (min–max)	35.4 (0.1–177.4)

**Table 4:** Descriptive information about laboratory parameters at the time of initial diagnosis.

Variables	Mean ± SD	Median (Min–Max)
B2 microglobulin	4.88 ± 3.67	3.55 (0.22–25.00)
Creatine	1.23 ± 1.02	0.92 (0.30–7.20)
Total protein	8.25 ± 1.96	7.80 (4.20–14.70)
Albumin	3.61 ± 0.86	3.63 (1.30–9.40)
Uric acid	6.42 ± 2.46	6.00 (1.33–20.0)
Calcium	9.63 ± 1.31	9.48 (6.90–16.0)
LDH	199.05 ± 83.81	189.50 (57.0–741.5)
CRP	20.18 ± 43.30	5.60 (0.0–373.0)
WBC	6995.20 ± 2622.60	6660.0 (1490.0–17,200.0)
Hb	11.16 ± 2.06	11.00 (6.10–16.30)
PLT	248,223.58 ± 88,552.50	242,500 (23,000–669,000)
MCV	91.50 ± 7.52	92.0 (62.0–124.0)
MPV	8.72 ± 1.45	8.85 (5.70–13.5)

CRP: C-reactive protein, Hb: Hemoglobin, LDH: lactate dehydrogenase, MCV: mean corpuscular volume, MPV: mean platelet volüme, PLT: platelet, WBC: white blood cell, SD: standard deviation.

**Table 5:** Patient OS comparisons.

Variables	2 Years %	5 Years %	Median (95% CI)	HR (95% CI)	*p*
Overall	87.0	70.1	NR		
Age					
≤65	87.5	72.5	NR	Ref.	0.439
>65	84.1	60.0	NR	1.30 (0.67–2.50)	
Gender					
Men	87.5	64.7	NR	Ref.	0.514
Women	85.9	74.9	NR	0.84 (0.50–1.42)	
Blood Type					
A	86.7	72.9	NR	Ref.	0.974
B	90.0	71.2	NR	1.13 (0.54–2.35)	
AB	85.0	68.0	NR	0.92 (0.35–2.40)	
0	86.9	68.3	NR	1.09 (0.60–2.00)	
ECOG					
0	92.4	75.8	NR	Ref.	<0.001
1	87.7	71.3	NR	1.41 (0.77–2.58)	
2 and above	60.8	52.1	NR	3.94 (1.77–8.78)	
Immunoglobulin Subtype					
IgA kappa	83.1	64.8	NR	1.11 (0.47–2.63)	0.632
IgA lambda	93.3	62.2	NR	0.53 (0.12–2.26)	
IgG kappa	88.0	68.0	NR	Ref.	
IgG lambda	86.8	72.0	NR	0.91 (0.44–1.86)	
Kappa Light Chain	80.7	62.4	66.5 (NE)	1.53 (0.78–2.99)	
Lambda Light Chain	75.8	75.8	NR	1.01 (0.30–3.39)	
ISS Stage					
Stage-1	88.5	82.2	NR	Ref.	0.041
Stage-2	84.6	70.7	NR	1.92 (0.84–4.38)	
Stage-3	83.9	61.0	66.5 (NE)	2.64 (1.21–5.74)	
R-ISS Stage					
Stage-1	92.1	89.5	NR	Ref.	0.077
Stage-2	91.7	75.6	NR	1.90 (0.74–4.89)	
Stage-3	76.7	56.2	NR	3.28 (1.13–9.51)	
Plasmacytoma					
MM with no plasmacytoma	90.2	66.4	NR	Ref.	0.049
EM-B	64.3	64.3	NR	1.79 (0.92–3.47)	
EM-S	91.7	78.6	NR	0.78 (0.44–1.38)	
Comorbidity					
Negative	86.4	65.4	NR	Ref.	0.053
Positive	87.4	78.3	NR	0.60 (0.35–1.01)	
Flow CD56 at First Diagnosis					
Negative	89.2	62.8	NR	Ref.	0.123
Positive	85.0	69.0	NR	1.07 (0.54–2.09)	
Unknown	89.8	79.6	NR	0.56 (0.25–1.25)	

Kaplan–Meier, log-rank test, *p* < 0.05 statistically significant; Note: Log-rank *p*-values were obtained after Kaplan–Meier estimation. Crude HRs were estimated using separate univariable Cox proportional hazards regression models for each variable. Abbreviations: ECOG: Eastern Clinical Oncology Group, CD: Cluster of differentiation, ISS: International Staging System, R-ISS: Revised International Staging System, NR: not reached, NE: not estimable.

**Table 6:** Patient PFS comparisons.

Variables	2 Years %	5 Years %	Median (95% CI)	HR (95% CI)	*p*
Overall	85.2	58.8	NR		
Age					
≤65	85.7	55.7	68.80 (NE)	Ref.	0.104
>65	91.0	73.5	NR	0.51 (0.22–1.17)	
Gender					
Men	87.9	58.9	NR	Ref.	0.196
Women	80.9	55.8	66.96 (NE)	1.36 (0.85–2.17)	
Blood Type					
A	88.0	66.9	NR	Ref.	0.562
B	78.6	57.4	NR	1.37 (0.69–2.72)	
AB	85.6	47.1	47.60 (NE)	1.45 (0.66–3.20)	
0	83.5	49.9	59.73 (44.39–75.07)	1.44 (0.82–2.50)	
ECOG					
0	87.5	52.2	66.99 (NE)	Ref.	0.699
1	82.9	59.6	NR	0.92 (0.56–1.50)	
2 and above	93.3	81.7	68.80 (14.48–123.11)	0.60 (0.18–1.99)	
Immunoglobulin Subtype					
IgA kappa	75.4	54.5	NR	1.41 (0.63–3.19)	0.471
IgA lambda	91.7	50.9	68.80 (NE)	1.20 (0.45–3.20)	
IgG kappa	86.3	58.8	NR	Ref.	
IgG lambda	88.6	52.5	NR	1.15 (0.59–2.23)	
Kappa Light Chain	71.0	41.4	51.03 (35.52–66.54)	1.88 (0.99–3.57)	
Lambda Light Chain	84.8	67.9	NR	0.87 (0.26–2.92)	
ISS Stage					
Stage-1	82.4	72.1	68.8 (NE)	Ref.	0.640
Stage-2	83.7	59.8	NR	0.93 (0.47–1.84)	
Stage-3	85.7	48.4	51.1 (NE)	1.25 (0.66–2.37)	
R-ISS					
Stage-1	82.6	70.1	66.96 (54.72–79.21)	Ref.	0.549
Stage-2	85.3	50.7	NR	1.04 (0.54–1.99)	
Stage-3	74.9	36.3	47.60 (25.42–69.77)	1.53 (0.67–3.47)	
Plasmacytoma					
MM with no plasmacytoma	87.5	60.7	NR	Ref.	0.742
EM-B	85.5	79.4	NR	0.91 (0.40–2.06)	
EM-S	82.5	54.0	NR	1.17 (0.71–1.91)	
Comorbidity					
Negative	84.3	51.5	60.26 (NE)	Ref.	0.103
Positive	86.3	66.2	NR	0.67 (0.42–1.09)	
Flow CD56 at First Diagnosis					
Negative	91.4	55.8	NR	Ref.	0.753
Positive	83.9	55.9	68.80 (NE)	1.27 (0.63–2.57)	
Unknown	83.4	59.4	NR	1.31 (0.63–2.73)	

Kaplan–Meier curve, Log-rank test, *p* < 0.05 statistically significant; Note: Log-rank *p*-values were obtained after Kaplan–Meier estimation. Crude HRs were estimated using separate univariable Cox proportional hazards regression models for each variable. Abbreviations: ECOG: Eastern Clinical Oncology Group, CD: Cluster of Differentiation, ISS: International Staging System, R-ISS: Revised International Staging System, NR: not reached, NE: not estimable.

**Table 7:** Multivariate Cox regression results on mortality risk for various clinical variables.

OS	Multivariate HR (95% CI)	*p*
ECOG		0.003
0	Ref.	
1	1.06 (0.51–2.20)	0.857
2 and above	3.78 (1.54–9.28)	0.004
ISS stage		0.034
Stage-1	Ref.	
Stage-2	2.25 (0.96–5.23)	0.060
Stage-3	2.93 (1.30–6.62)	0.010
Plasmacytoma		0.028
MM with no plasmacytoma	Ref.	
EM-B	2.46 (1.17–5.17)	0.017
EM-S	0.97 (0.48–1.95)	0.945

−2 Log Likelihood = 425.44, *p* < 0.001; Abbreviations: CI: confidence interval Note. HRs were mutually adjusted for ECOG performance status, ISS stage, and involvement status included simultaneously in the Cox proportional hazards regression model. ECOG: Eastern Clinical Oncology Group, ISS: International Staging System, MM: multiple myeloma (B: bone, S: soft tissue), OS: overall survival.

**Table 8:** Extramedullary involvement and its relationship with CD56.

CD56	MM with No Plasmacytoma	EM B + EM S	*p*
Negative	16 (11.9%)	22 (15.0%)	0.281
Positive	75 (56.0%)	90 (61.2%)	
Unknown	43 (32.1%)	35 (23.8%)	

Pearson Chi-square test, *p* < 0.05 statistically significant; Abbreviations: CD: cluster of differentiation, EM: extramedullary myeloma (B: bone, S: soft tissue), MM: multiple myeloma.

## Data Availability

For original data, please contact the corresponding author.
